# Recycled Cobalt from Spent Li-ion Batteries as a Superhydrophobic Coating for Corrosion Protection of Plain Carbon Steel

**DOI:** 10.3390/ma12010090

**Published:** 2018-12-27

**Authors:** Ali Rafsanjani-Abbasi, Ehsan Rahimi, Hossein Shalchian, Jalil Vahdati-Khaki, Abolfazl Babakhani, Saman Hosseinpour, Ali Davoodi

**Affiliations:** 1 Department of Materials Science and Metallurgical Engineering, Faculty of Engineering, Ferdowsi University of Mashhad, Mashhad 9177948974, Iran; ali.rafsanjani@gmail.com (A.R.-A.); rahimi.ehn@gmail.com (E.R.); hsh.metal@gmail.com (H.S.); Vahdati@um.ac.ir (J.V.-K.); babakhani@um.ac.ir (A.B.); 2 Department of Engineering and Architecture, University of Udine, Via Cotonificio 108, 33100 Udine, Italy; 3 Institute of Particle Technology (LFG), Friedrich–Alexander–Universität Erlangen-Nürnberg (FAU), Cauerstrasse 4, 91058 Erlangen, Germany

**Keywords:** Li-ion battery, recycling, superhydrophobic coating, electrodeposition

## Abstract

A new recycling and film formation scheme is developed for spent Li-ion batteries, which involves the combination of ascorbic-assisted sulfuric leaching and electrodeposition to fabricate a corrosion resistance superhydrophobic coating. The idea behind the simultaneous use of sulfuric and ascorbic is to benefit from the double effect of ascorbic acid, as a leaching reducing agent and as morphological modifier during electrodeposition. Quantum chemical calculations based on the density functional theory are performed to explain the cobalt-ascorbate complexation during the electrocristalization. The optimum parameters for the leaching step are directly utilized in the preparation of an electrolyte for the electrodeposition process, to fabricate a superhydrophobic film with a contact angle of >150° on plain carbon steel. The potentiodynamic polarization measurments in 3.5 wt % NaCl showed that boric-pulsed electrodeposited cobalt film has 20-times lower corrosion current density and higher corrosion potential than those on the non-coated substrate.

## 1. Introduction

The number and variety of secondary or rechargeable lithium-ion batteries (LIBs) have rapidly grown during the last two decades due to their favorable characteristics, such as high energy density, low self-discharge, high nominal voltage, long cycle life, lightweight design, and low cost [1,2]. The global market for LIBs is anticipated to reach more than 220 billion US dollars by 2024 [3]. Consequently, the generated waste from spent LIBs widely grows in the upcoming years. Also, more strict legislations regarding recycling of batteries increase the demand for developing new recycling methods of LIBs, to ensure clean environment, economic growth, and sustainable battery industry [4,5]. Nevertheless, often more than one methodology is required to achieve the desired economic and environmental goals, due to the complexity of the LIBs raw materials [6]. 

Hydrometallurgical extraction of metals, due to its low emission of dust and toxic gases, low-temperature treatments, low cost of infrastructures, and low consumption of energy, is the main route for recycling of LIBs [7,8]. The leaching of cathode active materials of LIBs has been widely studied using mineral acids, such as H_2_SO_4_, HCl, and HNO_3_ [9,10,11,12]. Since sulfuric and nitric acids are not strong enough for complete dissolution of Co^3+^, hydrogen peroxide is often considered as a potential reductive agent candidate for leaching of LiCoO_2_. The role of H_2_O_2_ in cathode active material leaching process is the reduction of Co^3+^ to Co^2+^, which has a better solubility in aqueous solutions [9,13,14]. Although, HCl and HNO_3_ almost successfully recover Co and Li to the leach liquor, they also introduce toxic gases to the environment and create risky working conditions. In addition, the emission of corrosive gases to the workplace could damage industrial instruments, which increases the subsequent expenses of the recycling process due to corrosion and failure problems.

To overcome mineral acid leaching challenges, recent studies have been focused on replacing mineral acids by organic ones, while maintaining the efficiency of leaching procedure [15,16,17]. The behavior of some organic acids, such as citric (C_6_H_8_O_7_) [17,18], DL-malic (C_4_H_6_O_5_) [19], oxalic (C_2_H_2_O_4_) [20], succinic (C_4_H_6_O_4_) [21], ascorbic (C_6_H_6_O_6_) [15,22,23], maleic (C_4_H_4_O_4_) [23], tartaric (C_4_H_6_O_6_) [24], formic (CH_2_O_2_) [25], aspartic (C_4_H_7_NO_4_) [26], and lactic (C_3_H_6_O_3_) [27] acids has been investigated for the leaching of LiCoO_2_ in recent studies.

In recent years, ascorbic acid (also known as Vitamine C) has become an increasingly important reducing agent for numerous transition metal ions and mononuclear complexes in chemical and biochemical systems [28]. Ascorbic acid is known as a mild reducing agent with a stable enediol structure, which can be oxidized by one or two electrons to a radical state or dehydroascorbic acid (C_6_H_6_O_6_) [29]. Hence, ascorbic acid is a potential alternative for hydrogen peroxide in leaching processes due to its remarkable reductive ability. Li et al., utilized ascorbic acid for the first time as both leaching and reductive agents to study the leaching of cobalt and lithium from spent LIBs with recovery efficiencies of 94.8% and 98.5%, respectively [15]. The proposed leaching reaction is as follows:

4C_6_H_8_O_6_ + 2LiCoO_2_ = C_6_H_6_O_6_ + C_6_H_6_O_6_Li_2_ + 2C_6_H_6_O_6_Co + 4H_2_O (1)

As it can be seen, the leaching products are cobalt and lithium complexes, in the absence of mineral acids. Thus, ascorbic acid has the potential of being a growth modifier in the deposition and synthesis of pure metals and metallic compound crystals. Modifying effect of ascorbic acid on crystal growth has been previously studied for different crystals, such as silver, gold, and ZnO [30,31,32].

A considerable number of reported recycling processes mainly focus on maximizing the recovery values of metal from spent batteries by optimizing the leaching parameters, such as time, temperature, solid to liquid ratio, acid concentration, reagent concentration, and rotation speed [33,34,35]. Only few studies have been carried out on the treatment and utilization of the obtained leaching solution [10,17,22,36,37,38]. In this research, we aim to develop a new recycling method to fabricate a superhydrophobic coating on plain carbon steel directly from leaching solution.

Superhydrophobic surfaces, defined as surfaces with contact angle higher than 150°, low sliding angle (SA < 10°), and low contact angle hysteresis (CAH). Such surfaces have attracted attention due to their desirable features, such as self-cleaning, high corrosion resistance, anti-icing, anti-frosting, drop splitting, and drop guiding properties [39,40,41,42,43,44,45]. To fabricate superhydrophobic surfaces, various methods have been developed, such as lithographic patterning, electrospinning, etching, layer-by-layer assembly, sol-gel, and chemical vapor deposition. However, most of these techniques have some restrictions and limitations, such as the need for special and expensive equipment and complicated process control, and often require special skills [46,47,48,49,50,51]. In contrast, electrodeposition technique has lately attracted researchers attention as a versatile method for the fabrication of the superhydrophobic surfaces, due to the fact that it is very convenient to carry out without special equipment [52,53].

This paper reports a novel recycling process for reductive leaching of spent Li-ion batteries, followed by electrodeposition, which results in the formation of a superhydrophobic coating on carbon steel. Sulfuric acid medium is selected as a primary leaching agent instead of HCl and HNO_3_ in order to have more economic recycling process and to overcome toxic and corrosive gases problems. In addition, low amounts of ascorbic acid, instead of the most common reducing agent (i.e., H_2_O_2_), are used to improve leaching efficiency. To benchmark the performance of ascorbic acid in comparison with H_2_O_2_, three groups of experiments are designed:

- sulfuric acid leaching without any reductive agent;
- sulfuric acid leaching in the presence of hydrogen peroxide; and,
- sulfuric acid leaching in the presence of ascorbic acid.

Also, the effect of most important parameters that alter the cobalt and lithium recoveries is studies. These parameters include leaching time and temperature, sulfuric acid concentration, reductive agent concentration, and pulp density. Eventually, the optimum condition of leaching measurements is directly employed for the preparation of an electrolyte for both direct and pulsed electrodeposition. Apart from being a reducing agent, ascorbic acid also modifies the morphology of the deposited crystals and thus alters the topography of the final deposited film. To evaluate the effect of ascorbic acid as a morphological modifier, its performance is compared with traditional growth modifier, boric acid. Finally, corrosion behavior of the electrodeposited film is investigated in 3.5 wt % NaCl using potentiodynamic polarization.

## 2. Materials and Methods 

### 2.1. Materials 

The cylindrical spent LIBs (commercial 18650 cell, from different manufacturers) from different manufacturers were collected for this study. The chemicals used for experiments, such as sulfuric acid, hydrochloric acid, hydrogen peroxide, and ascorbic acid were of the analytical grade. All of the solutions at specified concentrations were prepared in doubly distilled water.

### 2.2. Pretreatment of Spent LIBs

Spent batteries were manually dismantled according to safety precautions that are described in [54]. The cathode electrodes were thermally treated at 500 °C in an air atmosphere for 60 min to evaporate and decompose the organic binder. To obtain a homogenous fine powder, cathodic active material peeled from aluminum foils was milled for 10 min using planetary ball mill at room temperature at the rotation speed of 250 rpm. The obtained powder was characterized by X-ray diffraction (XRD, PW 1800, Philips, Amsterdam, The Netherlands) analyzer. 

### 2.3. Leaching Experiments

Leaching experiments were performed in a 250 mL glass reactor (solution volume 100 mL) immersed in a controlled water temperature bath that was equipped with a magnetic stirrer. A plastic cap was used at the reactor opening to reduce the loss of water due to evaporation. The factors influencing the recovery of lithium and cobalt from cathode active material were studied under different experimental conditions, including: H_2_SO_4_ concentration (0.5–2.5 M), H_2_O_2_ concentration (0–10 vol. % or 0–1.06 M), ascorbic acid concentration (0–0.25 M), temperature (30–90 °C), duration (15–180 min), and solid/liquid ratio (10–50 g·L^−1^). After each leaching, the obtained liquor was filtered and the solid residue remained on the paper filter. The filtrate was analyzed for cobalt and lithium content, using atomic absorption spectroscopy (AAS, PG 990, PG Instruments Ltd., Lutterworth, UK). In order to provide reference results for Co and Li accurate contents, 4 M hydrochloric acid at 85°C for 4 h was used to completely dissolve the spent LiCoO_2_ powders.

### 2.4. Electrodeposition

The optimum condition of leaching measurements from the previous section was directly employed to prepare the electrolyte for electrodeposition. A platinum ring (20 mm diameter) was employed as an anode for electrodeposition on the St-12 plain carbon steel plate as the cathode. Steel circular plates with 10 mm diameter were prepared while using the wire-cut machine. Electrical connection was made with a copper wires at the bottom of the St-12 circular plates and the whole assembly was cold mounted, leaving a 0.78 cm^2^ exposed surface area. The samples were wet-ground using SiC paper to 1200-grit finish and then washed in deionized water before electrodeposition.

**Figure 1:**
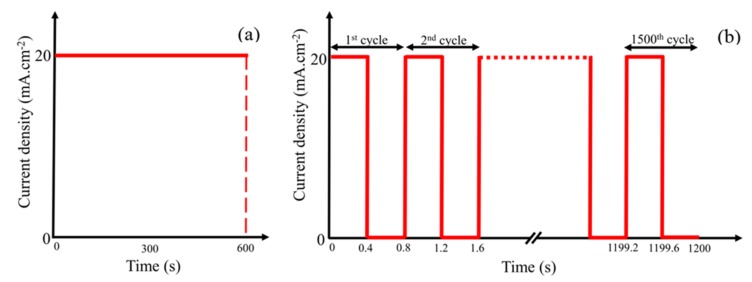
Current density-time curves of the electrodeposition procedure on plain carbon steel using optimum condition of leaching measurements as an electrolyte, (**a**) current control mode, and (**b**) pulsed current mode.

The electrodeposition process was carried out using CompactStat Ivium potentiostat instrument (Hampshire, UK) in the presence and absence of 0.5 M boric acid at an ambient temperature in either current control or pulsed current modes, which are schematically shown in Figure 1. In the current control mode, the applied current density on the St-12 plain carbon steel plate was kept constant at 20 mA·cm^−2^ for 600 s. The pulsed current mode was performed by 1500 deposition cycles, with each cycle consisting of 400 ms deposition time at a current density of 20 mA·cm^−2^ and 400 ms as the break time. 

### 2.5. Microstructural, Chemical and Simulation Analysis

The surface morphology of electrodeposited coatings was analyzed by scanning electron microscopy (SEM, Leo 1450 VP, Zeiss, Jena, Germany). Surface topography of deposited films was characterized by atomic force microscopy (AFM, Solver Next, NT-MDT Co., Goettingen, Germany) in the semi-contact mode with the scanning area of 20 μm × 20μm or 10 μm × 10 μm, a pixel resolution of 256 × 256, and scan frequency rate of 0.4 Hz. The surface chemical composition was analyzed using Fourier transformed infrared (Nicolet FT-IR—AVATAR 370, Thermo Fisher Scientific, Waltham, MA, USA) and X-ray diffraction (XRD) analyzer. For measuring and analysis of contact angle (CA), a drop shape analyzer device equipped with Nikon D3300 camera was used and the obtained imaged were processed by Image J (Drop Analysis LB-ADSA module) software (Version 1.X, LOCI, University of Wisconsin-Madison, Madison, WI, USA) using the Young-Laplass fitting algorithm. In order to measure the CA of the superhydrophobic surface, dynamic contact angle (DCA) was measured where the advancing and receding CAs were determined with 12 μL water droplet deposited on superhydrophobic sample (pulsed electrodeposition in the presence of boric acid). According to [55], the CA gives a measure of the stickiness of surface. The results demonstrated that the superhydrophobic cobalt coating has the advancing and receding angles of 152 ± 3° and 141 ± 2° (Δθ_CAH_ = 11 ± 1°), respectively.

In this study, the majority quantum chemical calculations of ascorbic acid and cobalt ascorbate molecules was carried out by the DMol3 module based on density function theory (DFT) in Materials Studio v 8.0 Accelrys Inc. software (San Diego, CA, USA). In addition, functional of Becke exchange plus Lee-Yang-Parr correlation (BLYP) was selected for the investigation of quantum chemical parameters in the geometrical optimization components. Total electrical density, the highest occupied molecular orbital (HOMO), and the lowest unoccupied molecular orbital (LUMO) were calculated for the adsorption of ascorbic acid. In addition, DMol3 density of states (DOS) was calculated by the Monkhorst-Pack k-point grid for a quick visualization of the electronic structure of cobalt-ascorbate molecule. DOS graphs can be used to explain parameters, such as the width of the valence band (E_v_), conduction band (E_c_), band gap energy (E_g_), and the number and intensity of the main features (HOMO and LUMO), and they are helpful in qualitatively interpreting the experimental spectroscopic results.

In order to investigate the adsorption mechanism of cobalt-ascorbate on Fe (100) plane surface during the electrodeposition process, Monte Carlo (MC) simulations were performed using the adsorption locator module and the COMPASS force field. The simulation was carried out in a simulation box (28.66 Å × 28.66 Å × 30.01 Å) with periodic boundary conditions.

### 2.6. Cyclic Voltammetry and Corrosion Behavior of the Coating

The cyclic voltammetry (CV) measurements were performed in optimum solution of leaching procedure at ambient temperature, from 200 to −1300 mV vs. open circuit potential (OCP) and the scan was reversed in −1300 mV with scan rate of 20 mV·s^−1^. Also, corrosion behavior of electrodeposited surfaces was examined in 3.5 wt % NaCl corrosive solution by means of OCP measurement and potentiodynamic polarization (PDP), with a scan rate of 1 mV·s^−1^, using CompactStat Ivium potentiostat.

## 3. Results

### 3.1. Optimization of Leaching Parameters

#### 3.1.1. Effect of Leaching Time

**Figure 2:**
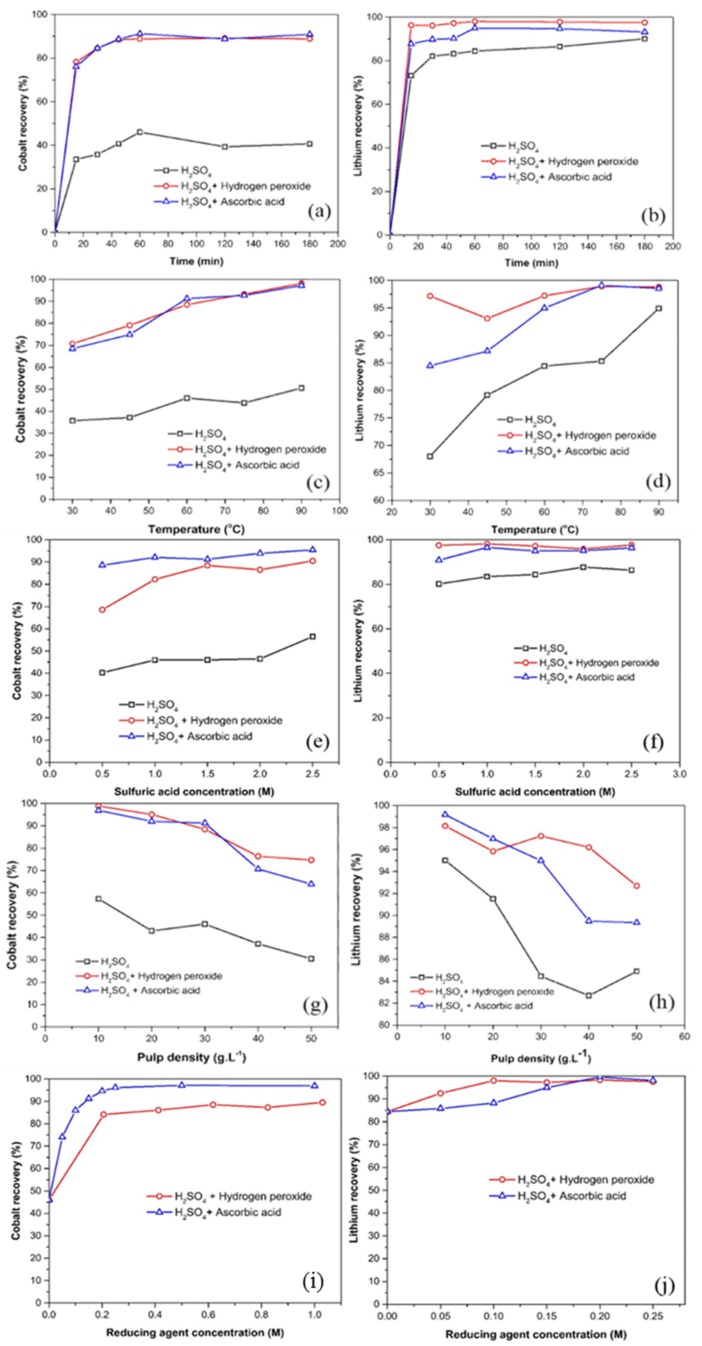
Effect of different parameters on Co and Li recovery by changing one parameter while keeping the other parameters constant at the center level. (**a**,**b**) Effect of time, T = 65 °C, H_2_SO_4_ = 1.5 M, C_6_H_8_O_6_ = 0.15 M, H_2_O_2_ = 0.636 M, and S/L = 30 g/L (**c**,**d**) Effect of temperature, Time = 60 min, H_2_SO_4_ = 1.5 M, C_6_H_8_O_6_ = 0.15 M, H_2_O_2_ = 0.636 M, S/L = 30 g/L (**e**,**f**) Effect of sulfuric acid concentration, Time = 60 min, T = 65 °C, C_6_H_8_O_6_ = 0.15 M, H_2_O_2_ = 0.636 M, S/L =30 g/L (**g**,**h**) Effect of pulp density, Time = 60 min, T = 65 °C, C_6_H_8_O_6_ = 0.15 M, H_2_O_2_ = 0.636 M, H_2_SO_4_ = 1.5 M (**i**,**j**) Effect of reducing agent concentration, Time = 60 min, T = 65 °C, H_2_SO_4_ = 1.5 M, S/L = 30 g/L.

Several batch leach tests were conducted to determine the optimum condition for recovery of lithium and cobalt. The recovery efficiency was determined using atomic adsorption spectroscopy, as was explained in the experimental section. During leaching experiments, the temperature was maintained at 65 °C and the pulp density was 30 g·L^−1^. Figure 2a,b shows variation in lithium and cobalt recoveries as a function of leaching time for the three studied leaching environments (only H_2_SO_4_, H_2_SO_4_ + hydrogen peroxide, or H_2_SO_4_ + ascorbic acid). Dissolution of lithium and cobalt is a solid-liquid reaction, which tends to progress at higher reaction times. After an initial incubation period, over 88% of cobalt and 93% of lithium was leached in 1.5 M sulfuric acid solution at the presence of 6 vol. % (0.636 M) of H_2_O_2_ or 0.15 M ascorbic acid, respectively. However, in the absence of these reducing agents, the leaching efficiency is relatively low, reaching only 46% of cobalt. It is worth noting that, for the three groups of leaching experiments, the percentage extraction increases with the time of reaction and the maximum dissolution is achieved after about 60 min. Consequently, further experiments were carried out at the constant duration of 60 min.

#### 3.1.2. Effect of Temperature

The effect of temperature on the amount of cobalt and lithium extraction in 1.5 M sulfuric acid at pulp density of 30 g·L^−1^, concentration of reducing agents 6 vol. % (0.636 M) H_2_O_2_ or 0.15 M ascorbic acid, and 60 min leaching time is plotted in Figure 2c,d, respectively. As can be seen in these figures, the leaching recovery of lithium and cobalt increases with the increase in the leaching bath temperature. In addition, while the recovery of lithium and cobalt in reductive dissolutions are, respectively, higher than 84% and 68% at 30 °C, their recovery increases to 94% and 88% at 60 °C, respectively. When the temperature for reductive leaching experiments was set to higher than 75 °C, over 97% of lithium and 92% of cobalt leached out, respectively.

#### 3.1.3. Effect of Sulfuric Acid Concentration

Recovery of valuable metals from spent LIBs has been widely performed in H_2_SO_4_ solution. Theoretically, the leaching reaction can be represented, as follows [56]:

4LiCoO_2_ + 6 H_2_SO_4_ = 2 Li_2_SO_4_ + 4 CoSO_4_ + 6 H_2_O + O_2_ (2)

Figure 2e,f shows the effect of sulfuric acid concentration on the leaching of cathode active material with 60 min leaching time, 30 g·L^−1^ solid to liquid ratio, and 60°C leaching temperature. Reducing agents concentration is maintained at 6 vol. % (0.636 M) and 0.15 M for hydrogen peroxide and ascorbic acid, respectively. The results show that, in the presence of reducing agents, for the whole range of the sulfuric acid concentration (0.5 M and 2.5 M), approximately 96% of lithium can be recovered. The percentage extraction of cobalt, in presence of hydrogen peroxide, increases gradually with increasing sulfuric acid molarity, approaching a constant value at 1.5 M sulfuric acid. Also, the extraction percentage of cobalt is relatively constant in the whole range of sulfuric acid concentration in the case of ascorbic acid. An acidic solution of 0.5 M sulfuric acid and 0.15 M ascorbic acid extracts 88% cobalt, while 1.5 M sulfuric acid solution containing 0.06 M hydrogen peroxide extracts the same amount of cobalt. In other words, when ascorbic acid is used as a reducing agent, sulfuric acid and reducing agent consumptions decrease by a factor of three and four, respectively. It is also observed that the reducing effect of ascorbic acid is stronger than hydrogen peroxide in the leaching of cobalt.

#### 3.1.4. Effect of Pulp Density

The leaching efficiency of cobalt and lithium with different pulp densities is plotted in Figure 2g,h, at a fixed condition of 1.5 M sulfuric acid concentration, 65 °C leaching temperature, and 1 h leaching time. Reducing agent concentration is maintained at 6 vol. % (0.636 M) and 0.15 M for hydrogen peroxide and ascorbic acid, respectively. As can be seen, the leaching efficiency decreases by increasing the pulp density. The reason for this negative effect of the pulp density in the recovery of cobalt and lithium is the reduction of the relative acid concentration as the pulp density increase. In other words, the ratio of reactants/pulp reaches below the stoichiometric value (Reactions (2), (3), and (5), *vide infra*) for the completion of reactions. It is therefore expected that the recovery trends to higher values if the reagents concentration, leaching time, and temperature increase in parallel with pulp density increment. Similar effects are observed for all three cases of study.

#### 3.1.5. Effect of Reducing Agent

Figure 2i,j illustrates the cobalt and lithium leaching efficiency as a function of reductive agent concentration at 65 °C, 1.5 M sulfuric acid concentration, and pulp density of 30 g·L^−1^ for 1 h leaching time, respectively. The effect of reducing agent is noticeable just for cobalt, because the lithium dissolution does not need to be leached reductively. As it can be seen, the presence of only 2 vol. % hydrogen peroxide increases the extraction percentage of cobalt considerably from 46% to 84%, which indicates the reducing role of hydrogen peroxide in leaching reactions. At higher concentrations of hydrogen peroxide, the leaching efficiency reaches to a constant value. In other words, 2 vol. % of hydrogen peroxide is sufficient for reaching the optimum reaction condition. H_2_O_2_ reacts with LiCoO_2_ in sulfuric acid containing media as follow [56]:

2 LiCoO_2_ + 3 H_2_SO_4_ + H_2_O_2_ = Li_2_SO_4_ + 2 CoSO_4_ + 4 H_2_O + O_2_ (3)

Hydrogen peroxide accelerates the leaching reactions of oxide particles by converting less soluble Co^3+^ into more soluble Co^2+^ with oxygen molecules released from hydrogen peroxide [14].

In the case of ascorbic acid, the behavior of the reducing agent is more obvious, since the addition of only 0.05 M ascorbic acid improves cobalt leaching efficiency by 28%. In addition, for the same molarities of ascorbic acid and hydrogen peroxide, recovery of cobalt in ascorbic acid containing solutions is obviously higher. For example, in the presence of 0.2 M ascorbic acid and 0.2 M hydrogen peroxide, the leaching efficiency of cobalt reaches about 94% and 84%, respectively. Similar to hydrogen peroxide, the key role of ascorbic acid in leaching process is the reduction of Co^3+^ to Co^2+^, which is more soluble in sulfuric acid. In this manner, ascorbic acid oxidizes to a radical state or dehydroascorbic acid (C_6_H_6_O_6_) [15]. The overall reaction can be described as:

2 Co^3+^ + C_6_H_8_O_6_ = 2 Co^2+^ + C_6_H_6_O_6_ + 2H^+^ (4)

in the presence of sulfate ions leaching reaction is represented, as follows:

3 H_2_SO_4_ + C_6_H_8_O_6_ + 2 LiCoO_2_ = Li_2_SO_4_ + 2 CoSO_4_ + 4 H_2_O + C_6_H_6_O_6_ (5)

Figure 2i,j shows that 1.5 M sulfuric acid solution, which contains only 0.25 M ascorbic acid, is strong enough for the near complete recovery of cobalt. The higher concentrations of ascorbic acid do not have a noticeable increment in cobalt leaching efficiency. These results indicate the presence of sufficient ascorbic acid for the reaction in concentrations as low as 0.25 M. The impressive effect of ascorbic acid that is demonstrated in this study reflects the high reducing ability of ascorbic acid. 

Summarizing of the results presented in Figure 2, it becomes evident that, for the same molarities of ascorbic acid and hydrogen peroxide, the extraction percentage of cobalt in ascorbic acid containing solution is obviously higher. Consequently, ascorbic acid could be used as a potential replacement of hydrogen peroxide in leaching experiments. Eventually, the optimum condition of leaching that results in more than 97% cobalt and 95% lithium recoveries are determined as: 1.5 M H_2_SO_4_ with 0.15 M ascorbic acid, a solid to liquid ratio of 30 g·L^−1^, a temperature of 90 °C, and leaching time of 60 min. This optimum set of parameters was directly employed to prepare electrolyte for both direct and pulsed electrodeposition in the following steps of this research.

### 3.2. Electrodeposition

#### 3.2.1. Cyclic Voltammetry and Simulation Studies

**Figure 3:**
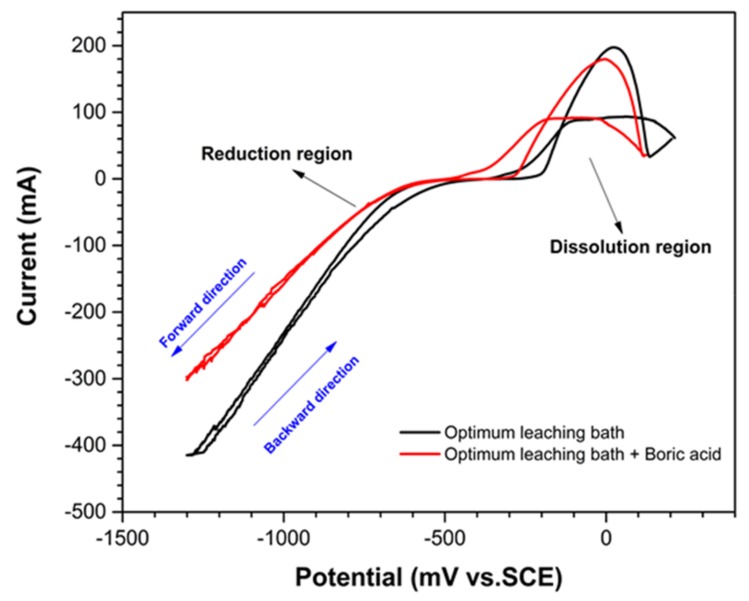
The cyclic voltammetry for electrodeposition onto plain carbon steel immersed in an optimum leaching bath of spent Li-ion batteries in presence and absence of boric acid. For a better clarity, the dissolution and reduction regions are marked.

Figure 3 shows the cyclic voltammograms for electrodeposition on the plain carbon steel substrate under the optimum reductive leaching condition, as described in the previous section, with or without the addition of boric acid. Boric acid (H_3_BO_3_) as a Lewis acid (pKa = 9.237) is added to the electrodeposition bath as an acidic catalyst to help the formation of hierarchical micro-nanostructured coating [57,58]. Tetrahydroxyborate anion (B(OH)^−4^) and hydronium cation (H_3_O^+^) are produced as the result of boric acid interaction with H_2_O. In fact, boric acid plays a leading role in controlling the morphology of micro or nanostructures during electrodeposition. In cyclic voltammograms in Figure 3, the cathodic reduction region (or electrodeposition process) includes both deposition and the side hydrogen evolution reactions. As can be observed from Figure 3, the potential of cathodic reactions shifts to the more negative values when boric acid is added to the electrodeposition bath. This potential reduction in the onset of the electrodeposition reactions can be explained by the capping and crystal growth modifying behavior of boric acid [59]. Essentially, boric acid molecules initially act as a buffering agent by attracting OH^−^ ions that formed near the substrate and result in H_2_ evolution. Subsequently, boric acid molecules adsorb onto the surface as neutral species, which strongly inhibit the reduction of Co^2+^ and shift cathodic reactions to more negative potentials [60]. 

A nucleation loop (or hysteresis loop) can be observed from the onset of cobalt reduction, from –680 ± 30 mV down to –990 ± 20 mV vs. SCE in the leaching bath without boric acid. This nucleation loop is due to the lower reduction current density when scanning in the forward direction when compared to that in the backward direction and it reflects the fact that a higher over potential is required for nucleation of cobalt complexes on plain carbon steel substrate. The addition of boric acid to electrodeposition bath effectively decreases the nucleation loop. 

**Figure 4:**
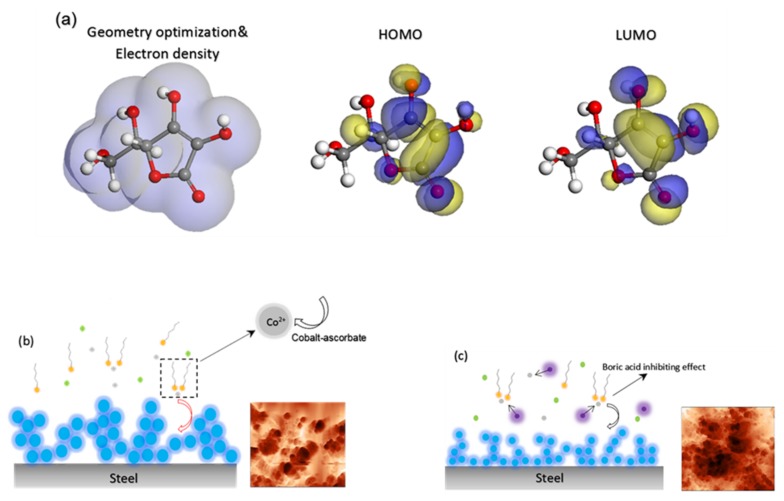
(**a**) Optimized geometry of ascorbic acid, highest occupied molecular orbital (HOMO) orbitals, and lowest unoccupied molecular orbital (LUMO) orbitals (**b**) schematic representation of ascorbic acid interaction with substrate and cobalt-ascorbate complex formation during electrocrystallization process, (**c**) crystal modifier action of boric acid molecule on crystal growth process.

To gain a better insight into the formation mechanism of hydrophobic complex during leaching and electrocrystallization processes, we performed quantum chemical calculations to reveal the ascorbic acid/steel surface interaction mechanism. Figure 4a shows the molecular structure of ascorbic acid molecule in its optimized geometry, together with total electron density, the energy of highest occupied molecular orbital (EHOMO), and the energy of lowest unoccupied molecular orbital (ELUMO), as calculated using DFT. Based on the frontier molecular orbital theory, a transition state can be formed between HOMO and LUMO orbitals of reactants during interaction and as a result of molecular structure evolution in the electrolyte [61]. Three main quantum chemical parameters of ascorbic acid are determined as: E_HOMO_= –6.91 eV, E_LUMO_= –1.73 eV, and band gap energy (ΔE = E_LUMO_ – E_HOMO_ = 5.18 eV). The E_HOMO_ parameter reflects the ability of a molecule to donate electrons and to form a bond with another molecule with an appropriate LUMO. The high energy value of HOMO demonstrates a higher bond formation probability [61]. Also, the high dipole moment (~5.49 D) indicates the tendency of ascorbic acid for forming a bond through electrostatic interactions [61]. During leaching process, the spent LiCoO_2_ initially dissolves with ascorbic acid in the form of a soluble C_6_H_6_O_6_Li_2_. The insoluble cobalt Co^3+^ ions are also reduced to soluble Co^2+^ by the ascorbic acid molecules. Eventually, oxidized ascorbic acid molecules react electrochemically with Co^2+^ ions, which leads to the formation of C_6_H_6_O_6_Co or cobalt-ascorbate (the more thermodynamically favorable form [15]), as shown schematically in Figure 4b. This interaction during leaching process can be explained by the following reaction [15]:

4C_6_H_8_O_6_ + 2LiCoO_2_ = C_6_H_6_O_6_ +C_6_H_6_O_6_Li_2_ +2C_6_H_6_O_6_Co +4H_2_O (6)

**Figure 5:**
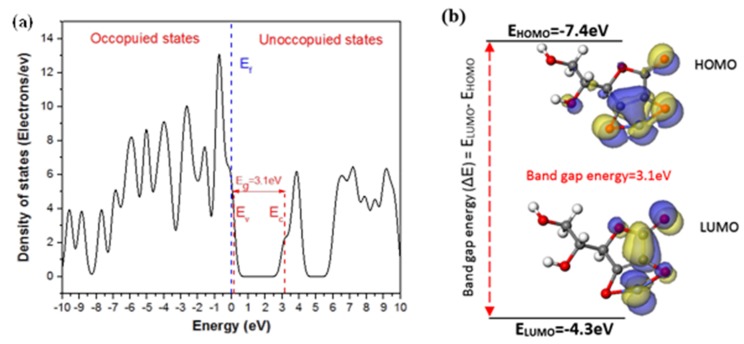
(**a**) The density of states (DOS) analysis of cobalt-ascorbate molecule calculated by DMol3, (**b**) HOMO, and LUMO orbitals of cobalt-ascorbate molecule with their corresponding band gap energy.

**Figure 6:**
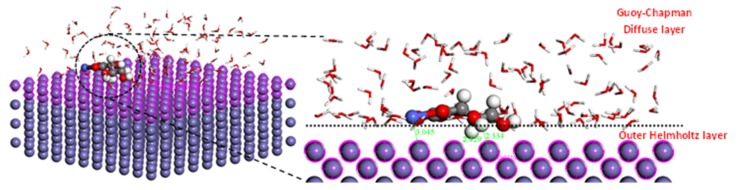
Top and slide views of cobalt-ascorbate molecule adsorption configuration in the presence of 100 water molecule before the onset of the electrocrystallization process.

To investigate the characteristics electronic structure of cobalt-ascorbate molecule, the density of states were used alongside other quantum chemical parameters, such as HOMO and LUMO. Figure 5 indicates the density of states computational analysis result of cobalt-ascorbate molecule. It can be observed that band gap of cobalt-ascorbate is predicated as ΔE = 3.1 eV, with a valance band of E_v_ = 0.11 eV and conduction band of E_v_ = 3.24eV. In addition, the high DOS distribution can be seen in the left part of Fermi level (occupied states), which is due to more presence of occupied electrons in comparison to in the right part of Fermi level (unoccupied states) [62]. Figure 5b shows HOMO and LUMO energy of cobalt-ascorbate molecule with values of –7.4 eV and −4.3 eV, respectively. DOS analysis and HOMO energy value in cobalt-ascorbate molecule indicate the high tendency of cobalt-ascorbate molecules to donate electrons to appropriate acceptor compound (steel surface in this case) before the completion of the electrocrystallization process [61]. Figure 6 shows the adsorption configuration of single cobalt-ascorbate on Fe (100) plane in the aqueous media, including 100 water molecules before electrocrystallization process. It can be observed from the slide view image that cobalt-ascorbate molecules have been adsorbed on Fe (100) surface with flat configuration, which represents a maximum coverage. Also, the values of adsorption energy and total energy are calculated to be −2737.3 kcal·mol^−1^ and −247.3 kcal·mol^−1^, respectively. For the adsorption of cobalt-ascorbate on steel surface, the cobalt-ascorbate molecules have to be transported from bulk solution (Gouy-chapman diffuse layer) toward the cathode surface (Fe (100)), near to inner Helmholtz layer, a step after which the stripping of the hydration sheath occurs at metal (Fe (100)/solution interface [63].

As it is shown in AFM maps in Figure 4c, in the presence of boric acid during the electrocrystallization process, a complex film with a fine structure is formed on the plain carbon steel substrate. The structure of this film is different from that formed in the absence of boric acid (Figure 4b). As was described earlier, the effect of boric acid on the film structure is related to its inhibiting or capping effect on crystal growth [64]. 

#### 3.2.2. X-ray Diffraction and FT-IR Analysis of the Deposited Films

**Figure 7:**
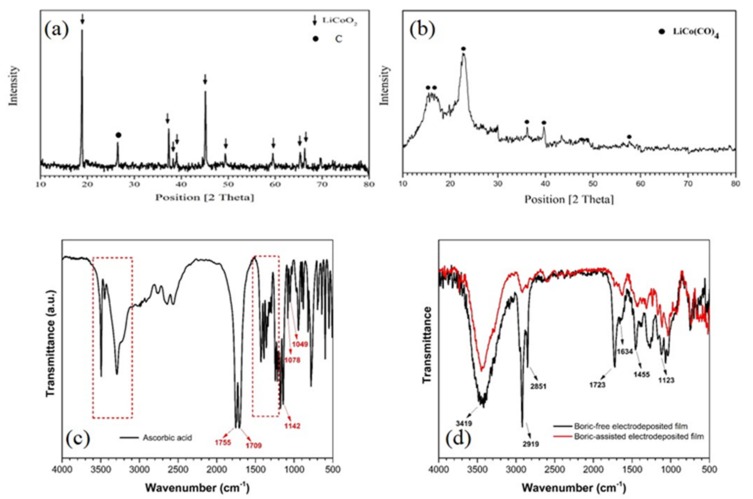
(**a**) XRD of the powder obtained from pretreatment of spent lithium-ion batteries (LIBs); (**b**) XRD of electrodeposited film onto plain carbon steel in optimum leaching bath of spent Li-ion batteries; (**c**) Fourier transformed infrared (FT-IR) spectra of ascorbic acid; and, (**d**) FT-IR spectra of electrodeposited coatings in presence and absence of 0.5 M boric acid.

The XRD analysis of homogenous fine powder that is obtained from pretreatment of spent LIBs shows the crystalline LiCoO_2_ phase, as shown in Figure 7a. Additionally, Figure 7b illustrates the XRD pattern of the electrodeposited film onto plain carbon steel in boric-free optimum leaching bath. The sharp peaks at diffraction angles of 15°, 22°, 36°, and 40° in the XRD pattern of the electrodeposited film are in good agreement with those that are given in No. 01-075-1366 data card of ICDD database for LiCo(CO)_4_. 

In order to investigate the interactions between different species, variations in chemical composition of the electrodeposited coating, and effect of ascorbic acid on leaching process, FT-IR spectroscopy was performed in the attenuated total internal reflection (ATR) mode. Figure 7c,d shows FT-IR spectra for pure L-ascorbic acid and electrodeposited film in the presence and absence of morphological modifier agent (i.e., boric acid). There are clear differences between the FT-IR spectra of the pure ascorbic acid and those deposited as films. These differences indicate that the structure of ascorbic acid undergoes changes when interacting with the plain carbon steel surface. 

The detailed assignment of the IR active vibrations on pure ascorbic acid is provided elsewhere [65]. The main observed bands are identified as the C=O and C=C stretching vibrations at 1755 cm^−1^ and 1709 cm^−1^, respectively, C–O–C stretching modes at 1142, 1078, and 1049 cm^−1^ [65,66], and the CH_2_ and the C–H deformation modes in the range of 1200 cm^−1^ –1500 cm^−1^. The presence of OH stretches strong bands in the range of 3100–3600 cm^-1^ indicates the adsorption of moisture on the sample [65]. Based on the FT-IR results, it is concluded that ascorbic acid molecules form ionic complexes with Co^2+^ ions with a hydrophobic chain during electrodeposition process [15].

As can be seen from Figure 7d, when the film is electrodeposited on the plain carbon steel, new bands appear in the corresponding FT-IR spectra. Absorption peak at 1634 cm^−1^ in electrodeposited films is related to (–C=C–) stretching vibration. The adsorption bands at 1123 cm^−1^ (C–O), 1723 cm^−1^ (C=O), and 3419 cm^−1^ (O–H) cm^-1^ observed for both boric-assisted and boric-free films are evidences for the presence of slight oxidation products and hydroxylation of the coating [43,67]. In contrast to the pure boric acid FT-IR spectrum, where barely any C–H stretching mode is observed, the C–H vibration modes at 2851 cm^−1^ and 2919 cm^−1^ are intense in the case of electrodeposited films, especially in the boric-free film. The sharp C–H bands in the FT-IR spectra of the films indicate a preferential ordering of the hydrophobic tail of deposited molecules, presumably protruding toward air away from the plain carbon steel substrate. The boric-assisted electrodeposited film has lower FT-IR peak intensities when compared with boric-free film, because, during the electrodeposition process, the boric acid creates a new complex with Co^2+^ ions [57,68]. 

#### 3.2.3. Corrosion Protection Behavior of the Electrodeposited Film

**Figure 8:**
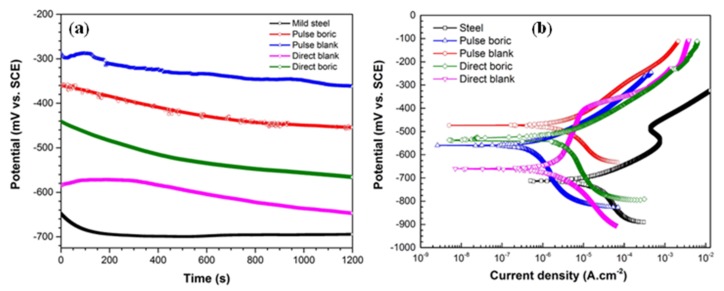
(**a**) Open circuit potential and (**b**) Potentiodynamic polarization curves of electrodeposited coatings immersed in 3.5 wt % NaCl with different conditions of electrodeposition.

In order to investigate the protective properties of the electrodeposited films under different deposition conditions in the corrosive media, we performed OCP and potentiodynamic polarization (PDP) measurements. The evolution of OCP for the plain carbon steel, and plain carbon steel with electrodeposited films in boric-assisted, and boric-free baths are evaluated in 3.5 wt % NaCl corrosive solution at 25 °C. As shown in Figure 8a, OCP of plain carbon steel reaches a steady-state condition after about 300 s, and clearly shows a lower value when compared to the OCP of the samples with electrodeposited films. Also, as can be seen in Figure 8a, pulsed electrodeposited coatings exhibit higher potential values in comparison with direct electrodeposited films. This difference between the OCP of pulsed and direct deposited films is attributed to a more non-conductive air pockets trapped in the electrodeposited micro-nanostructure [69,70,71], which will be discussed later. The high corrosion potential of pulsed electrodeposition samples demonstrates the effect of film deposition parameters on the susceptibility of plain carbon steel substrate to corrosion attack, which will be discussed in more details in the following. Potentiodynamic polarization curves of the plain carbon steel and electrodeposited films in 3.5 wt % NaCl solution at ambient temperature are presented in Figure 8b. The plain carbon steel and boric-free direct electrodeposited films show an activation control mechanism in their cathodic branch and a pseudo-passive behavior in their anodic branch. The pseudo-passive region for the boric-free direct electrodeposited film (in the range of −600 mV to −390 mV vs. SCE) is higher than that of the plain carbon steel (in the range of −565 mV to −470 mV vs. SCE). The corrosion protection that is offered by the presence of the electrodeposited film with micro-nanostructure is due to the presence of the cobalt complexes [70]. The corrosion potential and current density for all of the samples are extracted using the Tafel extrapolation method, and the results are provided in Table 1.

**Figure 9:**
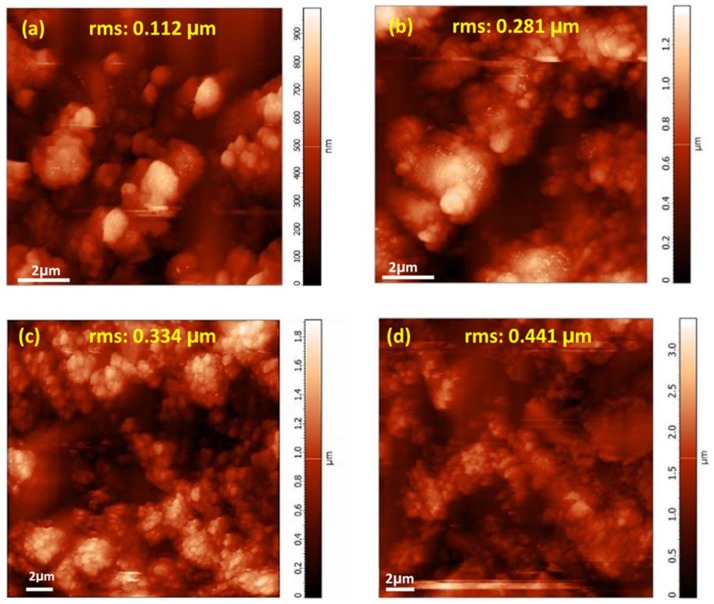
Atomic force microscopy of electrodeposited films; (**a**) boric-free direct electrodeposition, (**b**) boric-assisted direct electrodeposition, (**c**) boric-free pulsed electrodeposition, and (**d**) boric-assisted pulsed electrodeposition.

**Figure 10:**
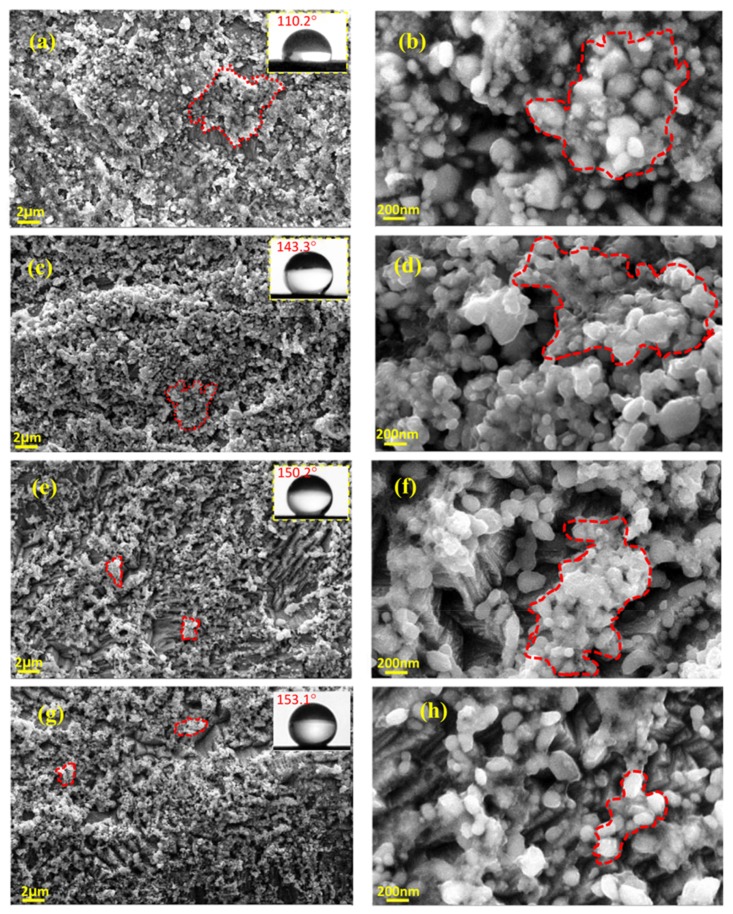
Scanning electron microscopy of electrodeposited coatings; (**a**,**b**) boric-free direct electrodeposition; (**c**,**d**) boric-assisted direct electrodeposition; (**e**,**f**) boric-free pulsed electrodeposition; and, (**g**,**h**) boric-assisted electrodeposition.

The effect of surface morphology and topography of the coatings on their wettability was investigated using atomic force and scanning electron microscopies. AFM images in Figure 9 show the morphology of the electrodeposited films that are prepared under different electrodeposition condition. As it is clearly demonstrated in these images, the electrodeposited films exhibit micro-nanostructures with an increase roughness in the order of: boric-free direct electrodeposition > boric-assisted direct electrodeposition > boric-free pulsed electrodeposition > boric-assisted pulsed electrodeposition. As can be seen in Figure 9 and Figure 10, pulsed electrodeposited coatings have a fine morphology distribution of microclusters along with nanoparticles and high root-mean-square roughness. The grooves that appear between the microcluster structures of the electrodeposited film provide potential sites for trapping air and consequently contribute to the superhydrophobicity of the electrodeposited film, as is depicted in Figure 10. The superhydrophobic films have high contact angles (>150°) due to the presence of air pockets and hydrophobic complexes [43,72]. The distribution of the fine structures contributing to the superhydrophobicity of the electrodeposited films is depicted in the SEM micrographs that are presented in Figure 9 and Figure 10. The hydrophobicity of the deposited films is expected to have a great effect on the corrosion kinetics, confirming the potentiodynamic polarization studies (Figure 8).

According to Figure 8b and Table 1, the superhydrophobic film that is fabricated by pulsed electrodeposition exhibits a higher corrosion potential and a lower current density than those of the hydrophobic film fabricated by direct electrodeposition method. 

**Figure 11:**
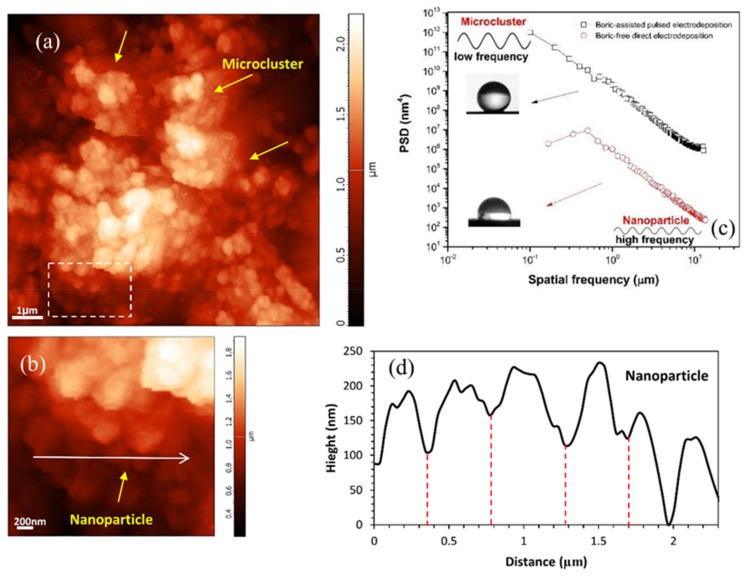
(**a**,**b**) Atomic force microscopy (AFM) image of microclusters and nanoparticles of superhydrophobic electrodeposited film; (**c**) power spectral density (PSD) versus the spatial frequency for boric-assisted electrodeposited film and boric-assisted direct electrodeposited film; and, (**d**) line profile of nanoparticles in a microcluster.

To further evaluate the differences between the morphology of the electrodeposited films, a power spectral density (PSD) analysis of AFM images are performed and the results are depicted in Figure 11. PSD is a frequency-based analysis of the surface roughness components, which provides more detailed information about the surface texture, as compared to conventional line scan analysis. This function can be calculated using the following equation [73]:

(7) $$\mathrm{PSD}\left(f\right)=\underset{A\to \infty }{\lim}\frac{1}{A}{\left|\int_{A}^{-}z\left(r\right)\exp\left(-2\pi \mathrm{if}.r\right)\mathrm{dr}\right|}^{2}$$

where z(r) is the height data of the surface roughness, A is the surface area of the measuring field, r is position vector, and f is the spatial frequency vector in the x−y plane.

It is observed that in the entire spatial frequency range, the PSD function of the superhydrophobic film fabricated by pulsed electrodeposition demonstrates a higher spectral roughness than that of the hydrophobic film (direct electrodeposition). The PSD function highlights the presence of microclusters in the superhydrophobic texture in comparison with the hydrophobic film, at low spatial frequency regions [64]. As was explained earlier, nanometric features in microclusters of the superhydrophobic film provide a suitable condition for increasing of air pockets in grooves with high roughness value in the high spatial frequency [74]. Figure 11b,d shows AFM image and line profile of nanoparticles in a microcluster on superhydrophobic electrodeposited film, respectively. This specific morphology of the electrodeposited film accommodates pockets of trapped air within the microstructure of the electrodeposited film and increases the Laplace pressure, which leads to lower contact angle [53]. Furthermore, air pockets that are trapped in micro-nanostructure of superhydrophobic films behave as an isolative barrier, which limits the electron transfer in the electrochemical reactions during corrosion and thus enhances the corrosion resistance of the coating. 

## 4. Conclusions

A superhydrophobic coating with a contact angle of >150° was fabricated over plain carbon steel by combination of ascorbic-assisted sulfuric leaching of spent Li-ion batteries, followed by electrodeposition. The optimized parameters of leaching solution with more than 97% cobalt and 95% lithium recoveries were as follows: 1.5 M H_2_SO_4_ with 0.15 M ascorbic acid, a solid to liquid ratio of 30 g·L^−1^, a temperature of 90 °C, and leaching time of 60 min. The optimized leaching solution was directly employed as an electrodeposition bath in both direct and pulsed electrodeposition to provide a protective coating on carbon steel. X-ray diffraction and FT-IR spectroscopy approved the presence of cobalt-containing complexes such as LiCo(CO)_4_ in the electrodeposited coating. Also, boric-assisted pulsed electrodeposited coating showed higher corrosion potential and lower current density than that of boric-free and direct electrodeposited films. In addition, SEM and AFM analysis showed that large grooves between the microcluster structures of the electrodeposited film provide a good condition for trapped air, which can reduce the susceptibility of plain carbon steel to corrosion. Likewise, potentiodynamic polarization measurements demonstrated that boric-pulsed electrodeposited cobalt film have 20 times lower corrosion current density (0.5 < 10 µA·cm^−2^) and higher corrosion potential (−557 > −712 mV vs. SCE) than substrate. 

## Figures and Tables

**Table 1:** Potentiodynamic polarization parameters of electrodeposited coatings in different conditions.

Surface	i_corr_ (µA·cm^−2^)	E_corr_ (mV vs. SCE)	b_a_ (mV/decade)	b_c_ (mV/decade)
Boric-assisted pulsed electrodeposition	0.5 ± 0.1	−557 ± 14	52 ± 2	126 ± 2
Boric-free pulsed electrodeposition	1 ± 0.1	−471 ± 15	60 ± 3	78 ± 4
Boric-assisted direct electrodeposition	1.4 ± 0.2	−534 ± 10	75 ± 2	83 ± 5
Boric-free direct electrodeposition	1.6 ± 0.2	−662 ± 12	210 ± 4	138 ± 3
Plain carbon steel	10 ± 0.5	−712 ± 10	72 ± 3	128 ± 4
